# ZB716, a steroidal selective estrogen receptor degrader (SERD), is orally efficacious in blocking tumor growth in mouse xenograft models

**DOI:** 10.18632/oncotarget.24023

**Published:** 2018-01-08

**Authors:** Shanchun Guo, Changde Zhang, Melyssa Bratton, Madhusoodanan Mottamal, Jiawang Liu, Peng Ma, Shilong Zheng, Qiu Zhong, Lin Yang, Thomas E. Wiese, Yong Wu, Matthew J. Ellis, Margarite Matossian, Matthew E. Burow, Lucio Miele, René Houtman, Guangdi Wang

**Affiliations:** ^1^ Department of Chemistry, Xavier University of Louisiana, New Orleans, LA 70125, USA; ^2^ RCMI Cancer Research Center, Xavier University of Louisiana, New Orleans, LA 70125, USA; ^3^ College of Pharmacy, Xavier University of Louisiana, New Orleans, LA 70125, USA; ^4^ College of Pharmacy Chongqing Medical and Pharmaceutical College, University Town, Chongqing, 401331, China; ^5^ Department of Internal Medicine, Charles Drew University, Los Angeles, CA 90059, USA; ^6^ Lester and Sue Smith Breast Center, Baylor College of Medicine, Houston, TX 77030, USA; ^7^ Section of Hematology & Medical Oncology, Tulane University School of Medicine, New Orleans, LA 70112, USA; ^8^ Department of Genetics, Louisiana State University Health Sciences Center, New Orleans, LA 70112, USA; ^9^ Nuclear Receptor Group, PamGene International, 5211HH Den Bosch, The Netherlands

**Keywords:** steroidal oral SERD, breast cancer, estrogen receptor mutant, Y537S, bioavailability

## Abstract

Advances in oral SERDs development so far have been confined to nonsteroidal molecules such as those containing a cinnamic acid moiety, which are in earlystage clinical evaluation. ZB716 was previously reported as an orally bioavailable SERD structurally analogous to fulvestrant. In this study, we examined the binding details of ZB716 to the estrogen receptor alpha (ERα) by computer modeling to reveal its interactions with the ligand binding domain as a steroidal molecule. We also found that ZB716 modulates ERα-coregulator interactions in nearly identical manner to fulvestrant. The ability of ZB716 to inhibit cell growth and downregulate ER expression in endocrine resistant, ERα mutant breast cancer cells was demonstrated. Moreover, in both the MCF-7 xenograft and a patient derived xenograft model, orally administered ZB716 showed superior efficacy in blocking tumor growth when compared to fulvestrant. Importantly, such enhanced efficacy of ZB716 was shown to be attributable to its markedly higher bioavailability, as evidenced in the final plasma and tumor tissue concentrations of ZB716 in mice where drug concentrations were found significantly higher than in the fulvestrant treatment group.

## INTRODUCTION

Selective estrogen receptor downregulators (SERDs) are a class of endocrine agents that act both as estrogen receptor (ER) antagonists and ER degraders. Currently the only FDA approved SERD is fulvestrant, originally indicated for breast cancer progressing after tamoxifen or aromatase inhibitor (AI) treatment, but recently approved for first line endocrine therapy [1–4]. While fulvestrant has proven clinically effective with manageable adverse side effects, the drug is well known for its poor bioavailability [5]. It can only be administered as a monthly intramuscular injection and is believed to have limited drug exposure and ER turn-over in patients [6–7]. In the second and greater line setting, the low bioavailability of fulvestrant and its slow action may in particular contribute to limited efficacy because the endocrine-resistant tumor requires an even higher drug exposure [8–11]. In the first line setting fulvestrant's route of i.m. administration and the long time it takes to reach steady state drug concentration in systemic circulation may limit its wider clinical application. Orally bioavailable SERDs, therefore, are highly desirable with potential to bring substantial clinical benefits to patients in need of endocrine therapy, especially in the advanced metastatic setting.

Advances in oral SERDs development so far have been confined to nonsteroidal molecules among which the most promising SERDs are those containing a cinnamic acid moiety, believed to be a critical structural feature conferring SERD-like properties [12]. Several oral SERDs have entered clinical trials since 2014, including GDC-0810 and AZD9496 [13–14]. These compounds have shown promising preclinical results, including strong antiestrogenic activity, ER downregulating efficacy comparable to fulvestrant, and favorable pharmacokinetic profiles. Their clinical performance, however, has yet to be proven. Notably, the dose chosen for phase II trial of GDC-0810 [15], at 600mg per day, is indicative of its high concentration requirement to be therapeutically effective and/or relatively modest bioavailability in patients demonstrated in the phase I trial [13]. Such high dose requirement may add to its potential gastrointestinal tolerability challenge in subsequent clinical development [16].

**Figure 1:**
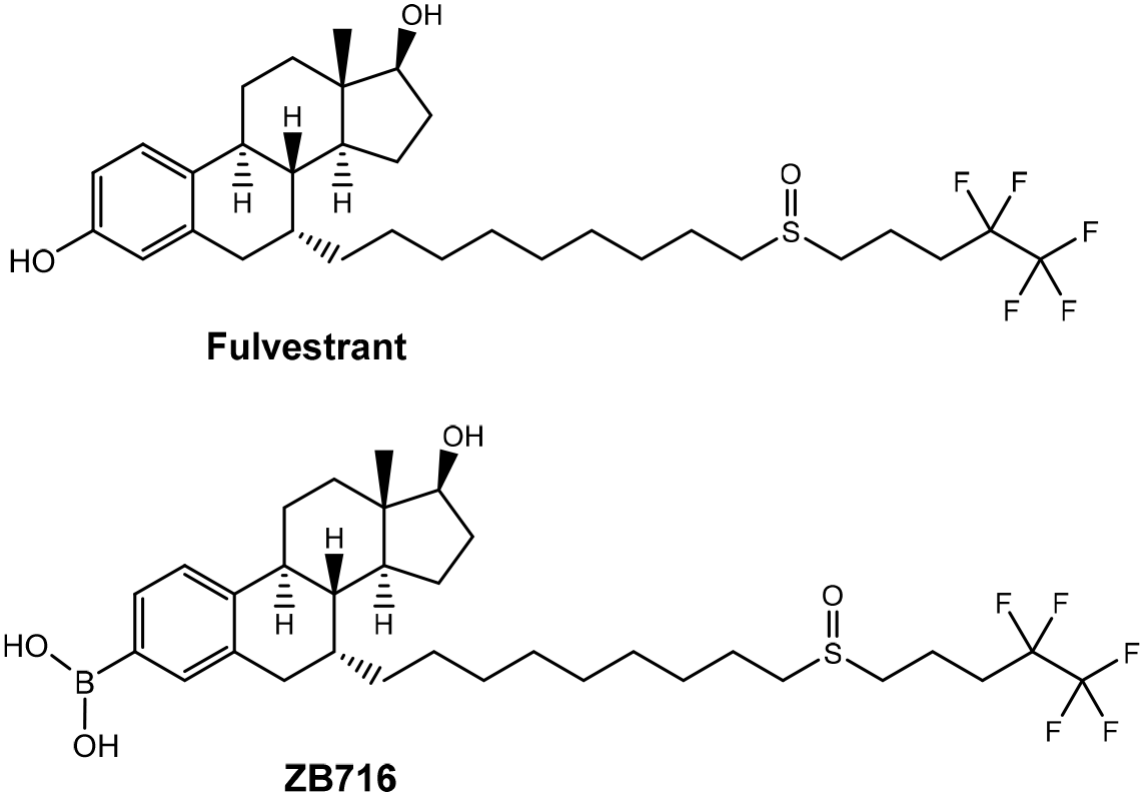
Structures of ZB716 and fulvestrant

ZB716 is an orally bioavailable SERD that is structurally analogous to fulvestrant but modified by replacing the 3-hydroxyl group with a boronic acid moiety [17] (Figure 1). The rationale for this chemical design was based upon previous work [18–20] where boronic derivatives of phenolic compounds were found to confer much greater oral bioavailability by avoiding first-pass metabolism. We showed that ZB716 largely retained the pharmacological properties of fulvestrant but with vastly improved oral bioavailability [17]. Indeed, ZB716 afforded over 10 times higher plasma peak concentrations after single-dose oral gavage than same-dose fulvestrant administered subcutaneously in mice. Here, we first examine the binding of ZB716 to ER alpha (ERα) in comparison with fulvestrant to better understand the remarkable similarity between the binding affinities of the two molecules as reported previously. Next, efficacy studies demonstrate that orally administered ZB716 is effective in blocking the growth of MCF-7 derived xenograft tumor and patient derived ER+ breast tumor in mice. Compared to fulvestrant, ZB716 demonstrates significantly improved tumor tissue exposure to drug, consistent with enhanced drug levels in systemic circulation. Pharmacokinetic studies in both mice and rats confirm high oral bioavailability and sustained steady-state drug concentration in blood stream. These remarkable improvements over fulvestrant make ZB716 an excellent steroidal oral SERD with the potential to enter clinical trials as first of its kind. It is also anticipated that given its known mode of action and adverse side effects likely similar to fulvestrant, ZB716 may offer a clearer path to the clinic than the nonsteroidal SERDs undergoing clinical trials.

## RESULTS

### The binding of ZB716 to ER as studied by molecular modeling

**Figure 2:**
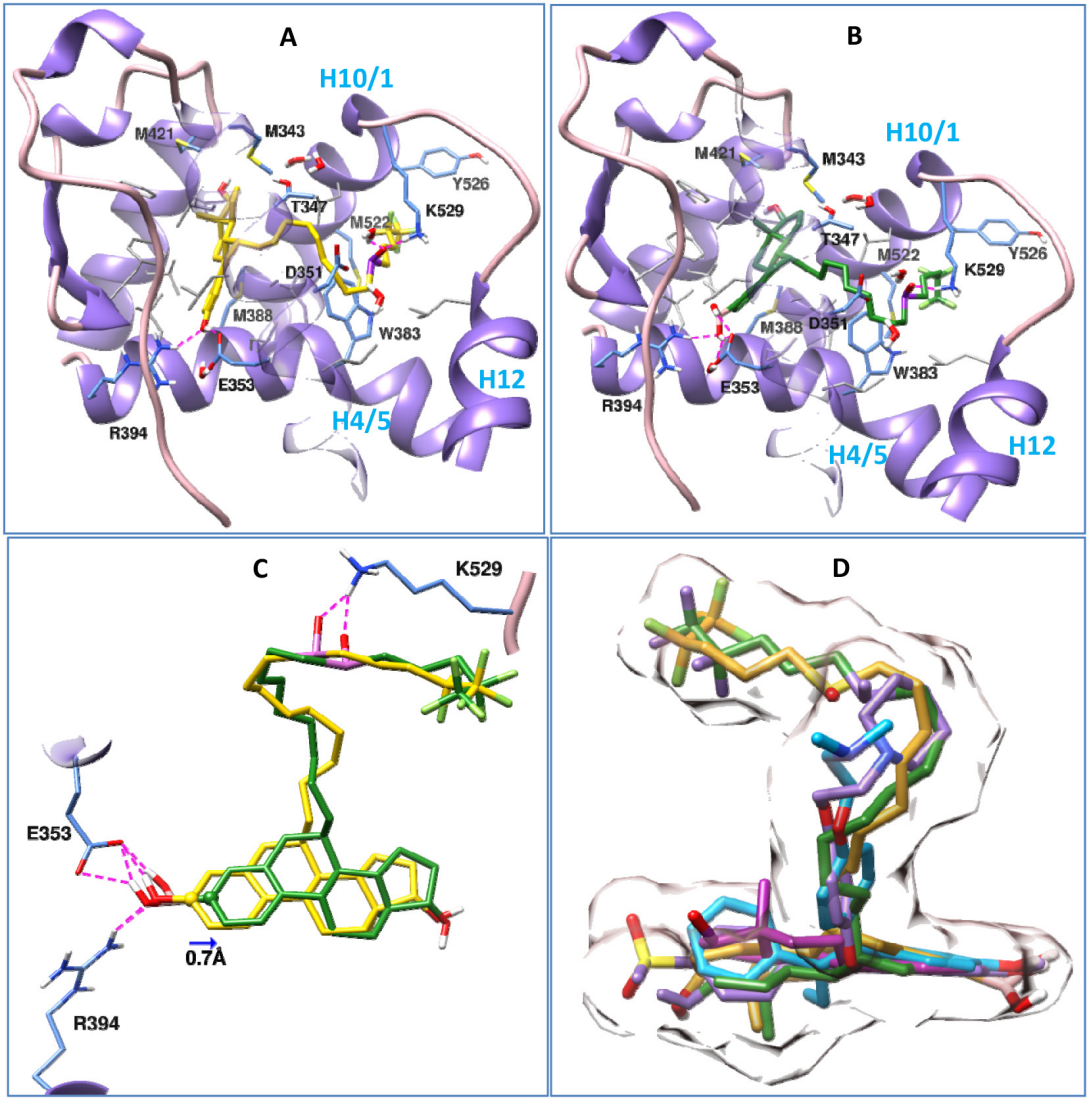
Binding postures of Fulvestrant, ZB716 and three crystal ligands in the antagonistic binding site of ERα Important amino acids in the binding pockets are shown in stick models, among them the hydrophobic residues are shown in grey, and ERα is depicted in ribbon model. Both Fulvestrant and ZB716 form hydrogen bond with Glu353, Arg394 and Lys529. Subset of Figure 2 are **(A)** Fulvestrant in complex with ERα, **(B)** ZB716 in complex with ERα, **(C)** superposition of Fulvestrant (yellow) and ZB716 (green) in the binding pocket of ERα, and **(D)** superposition of Fulvestrant (yellow), ZB716 (green), 4-hydroxy tamoxifen (cyan), estradiol (magenta) and the crystal ligand in 2ayr.pdb (purple) in the antagonistic binding pocket of ERα. Surface representation of fulvestrant and the crystal ligand in 2ayr is shown to outline the shape of the binding pocket.

Having found both fulvestrant and its boronic acid derivate (ZB716) bind ER_α_ with comparable binding affinities (EC_50_ 3.8 nM vs EC_50_ 4.1 nM)[17], we compared the binding of fulvestrant and ZB716 to ER_α_ in the antagonistic conformation using molecular docking method. Our study shows (Figure 2) that both fulvestrant and ZB716 can bind to the antagonistic ligand binding site of ERα with high compatibility. As seen in the crystal structures of ER in complex with other antagonists [21–24], the steroidal moiety of the fulvestrant molecule (Figure 2A) binds exactly in the same region as the main scaffold of the antagonistic ligands, which is almost identical to the binding of estradiol to ERα [25]. The fluropentyl sulphinyl containing long linker chain was found to protrude through the opening region constituted by helix 10/11, helix 12 and helix 4/5. Placement of the linker chains in the opening region of helix 10/11, helix 12 and helix 4/5 of ERα was also seen for other antagonists, like 4-hydroxytamoxifen (3ert.pdb), [6-hydroxy-2-(4-hydroxyphenyl)-1-benzothien-3yl] [4-(2-phrrolidin-1-ylethoxy)phenyl]methanonone (2r6y.pdb) and the crystal ligand used in the present study (2ayr.pdb). At one end of the binding pocket, the 3-hydroxyl group of the fulvestrant formed hydrogen bonds with Glu353 and Arg394. These hydrogen bonds are conserved in many ER-antagonist and ER-agonist complexes, including 4-hydroxy tamoxifen and estradiol [21, 23–25]. The hydroxyl group on the cyclopentane ring of fulvestrant reached out to the other end of the binding pocket and got solvated by water molecules. The four hydrocarbon rings of the estradiol moiety of fulvestrant formed van der Waals contact with several hydrophobic residues as shown in Figure 2A. The sulfonyl group of the linker forms a hydrogen bond with Lys529 and enhances the passing of the linker through the opening region.

ZB716 (Figure 2B) also binds to ERα in a similar manner as fulvestrant. Though the hydroxyl group was replaced with a boronic acid group, the placement of the estradiol moiety of ZB716 in the binding pocket and hydrogen bond formation with Glu353 and Arg394 were observed as in fulvestrant (Figure 2A). This was achieved because of the smaller size of boron, and it required only a slight (0.7Å) shifting of the molecule along the binding pocket as seen in the superimposed structures of fulvestrant and ZB716 (Figure 2C). The docking scores for both drugs are comparable at −10.85 and −11.28 kcal/mol for fulvestrant and ZB716, respectively. Similarly the free energies of binding obtained by MMGB/SA calculations are also comparable to each other and they are −181.39 and −183.41 kcal/mol for fulvestrant and ZB716, respectively. A comparison of the binding of fulvestrant and ZB716 with the crystal structure of antagonists in complex with ERα (Figure 2D) showed a similar binding pattern.

### Compound-induced modulation of ERα-coregulator interaction

Molecular modeling shows highly similar ERα-binding modes of fulvestrant and ZB716. This suggests that upon binding to the ligand binding pocket both compounds induce a similar ligand-binding domain (LBD) conformation and affinity for coregulator proteins. To test this, we measured binding of ERα LBD to a peptide microarray containing 154 individual (CoR-) NR-boxes of a set of 60+ coregulators in absence (apo) or presence of compound.

**Figure 3:**
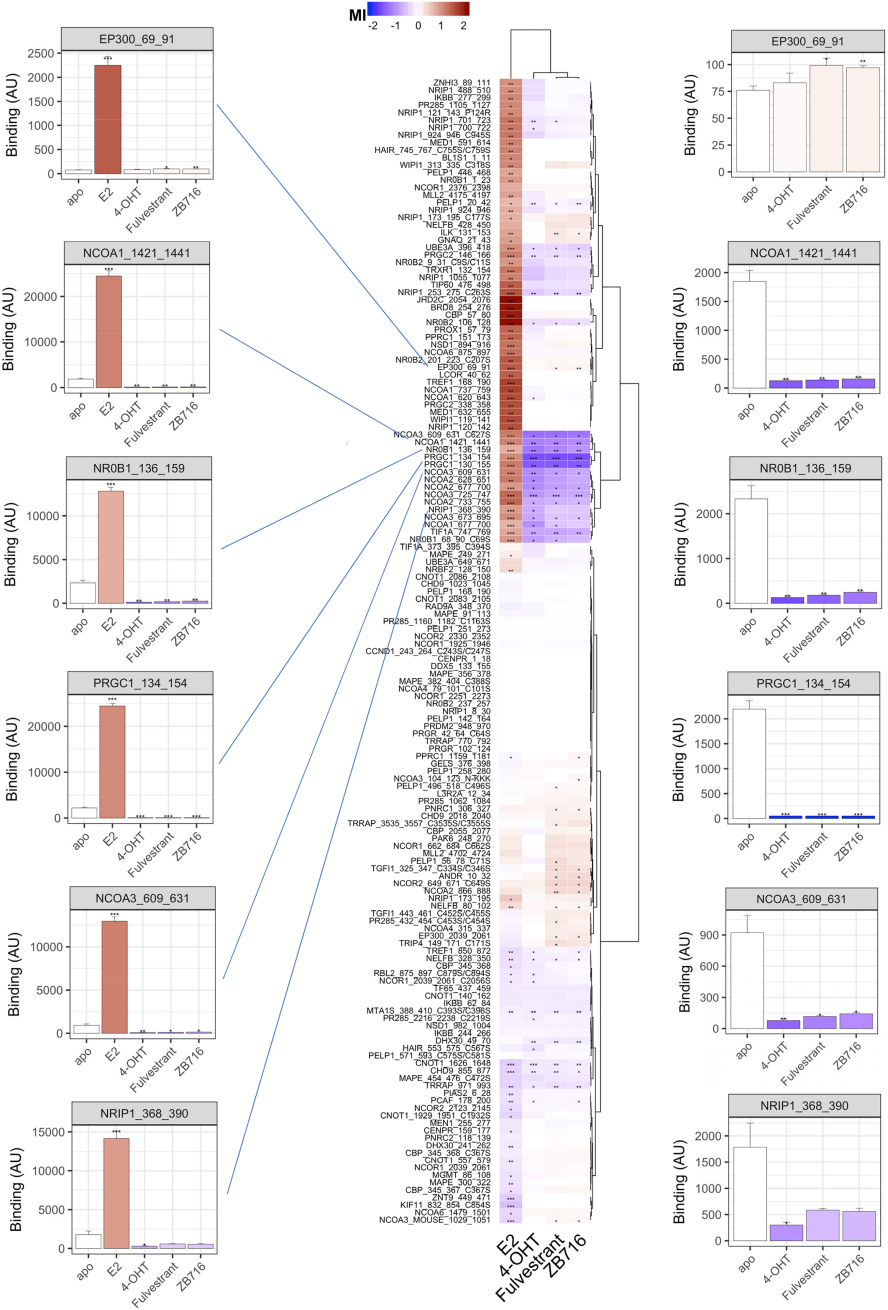
Modulation of ERα-coregulator interaction by 17-β-Estradiol (E2), 4-hydroxy-tamoxifen (4-OHT), fulvestrant, and ZB716 Compound effects are indicated by the modulation index (MI), i.e. compound-induced log-fold change of ERα-LBD interaction with peptides representing individual coregulator-derived binding motifs. Enhancement of binding are indicated in red while peptide displacement is indicated in blue. Compound and interaction (dis)similarities are visualized by Hierarchical clustering (Euclidean distance, Ward’s). Bar graphs display ER binding (mean +/− S.E.M., Arbitrary Units fluorescence) in the absence (apo) or presence of compound. The bar color represents the MI. Significance of the modulation is indicated (^*^p<0.05; ^**^p<0.01; ^***^p<0.001, Student's t-Test vs. apo).

Per compound, the effect was assessed by calculation of the modulation index (MI), i.e. compound-induced log-fold change of binding, for the interaction of ERα with each individual coregulator motif. As shown in the heat map of Figure 3, this results in a modulation profile (column) per compound of 154 interactions that are either potentially enhanced (red) or decreased (blue). To visualize (dis)similarities between compounds (and coregulator interactions) we applied hierarchical clustering.

When using the agonist 17-β-estradiol (E2, bottom row) the majority of affected interactions show enhancement of ERα binding, as illustrated by the cluster of motifs at the top half of the heat map. This cluster is highly enriched for motifs from coactivator proteins, i.e. enhancers of transcription, as can be expected from receptor stimulation with an agonist. The lower sub-cluster of E2-enhanced interactions largely consists of motifs from the members of the Nuclear Receptor Coactivator (NCOA) family. Alternatively, the interaction of ERα with these same motifs is strongly reduced upon incubation with antagonists 4-hydroxy-tamoxifen (4-OHT), fulvestrant or ZB716. The full modulation profiles are clearly differential between compounds with agonist and antagonist behavior. Moreover, whereas upon additional comparison of the antagonists we observe strong overlap in modulation of receptor-coregulator interactions and fulvestrant and ZB716 are virtually identical, there are clusters of interactions that are differentially modulated by 4-OHT. To illustrate the observations as described above, we have selected ER interactions with some well documented coregulators in absence (apo) or presence of indicated compound as bar graphs. On the left, E2 strongly enhances binding, suggesting strong recruitment of these coregulators to the locus of target genes. Alternatively, while the SERDs and SERM appear to do the opposite, the y-axis scale is largely dominated by E2. We plot the same interactions on the right of the heat map with the E2 data to enable further comparison of these compounds. While 4-OHT, fulvestrant and ZB716 act highly similarly, some of the interactions display a moderate differential behavior for the SERDs vs. the SERM. A similar visualization (with or without E2) for each individual motif on the array is provided in supporting information (Supplementary Figure 1).

These modulation profiles strongly suggest subtle differences in the ERα conformation as induced upon binding of 4-OHT and fulvestrant, and more importantly, confirm that fulvestrant *in vitro* pharmacology is completely preserved in ZB716 despite the introduction of the boronic acid moiety, as was predicted by molecular modeling.

### ZB716 inhibits cell growth and degrades ER in MCF-7 and in T47D/Y537S breast cancer cells

**Figure 4:**
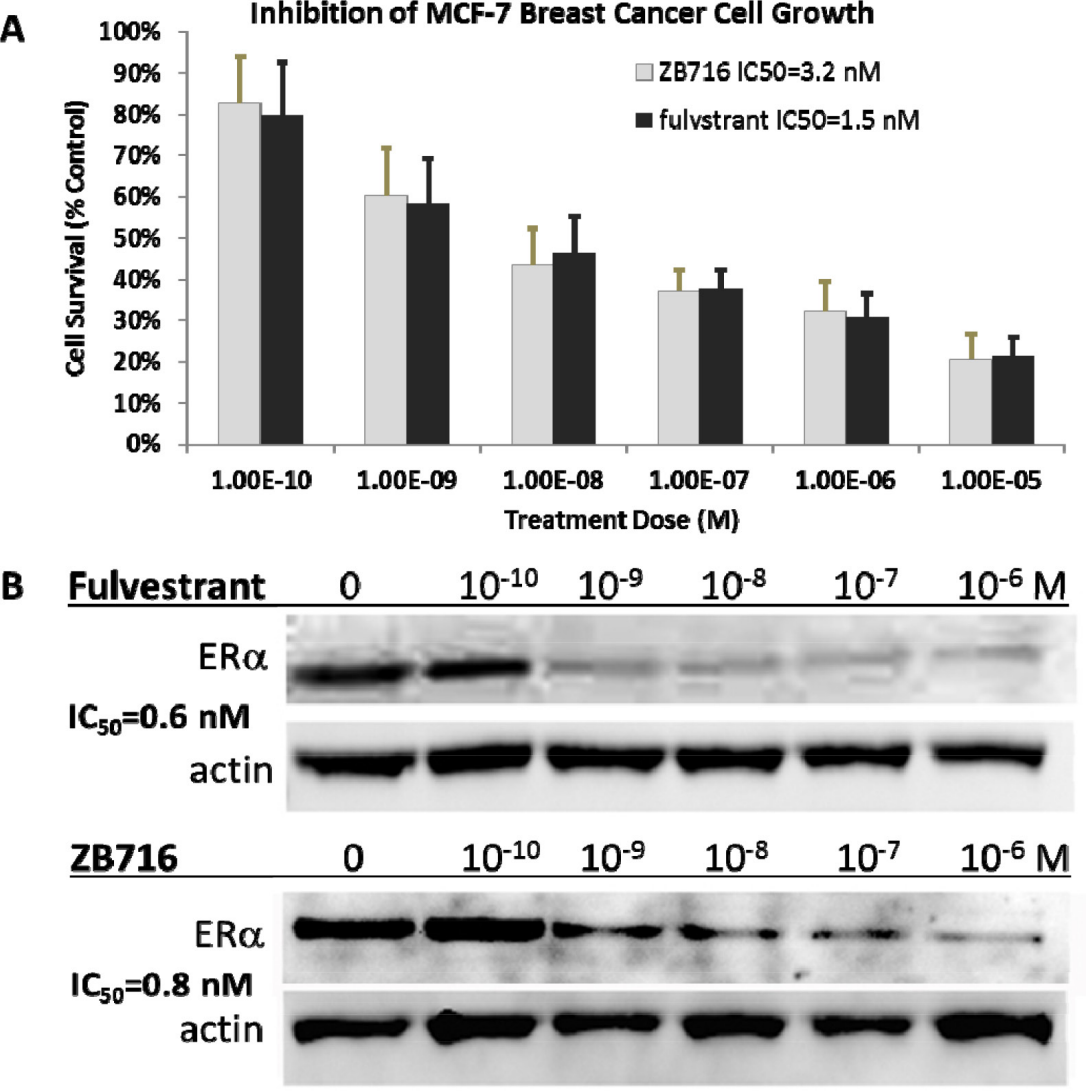
**(A)** MCF-7 breast cancer cells were treated with increasing doses of ZB716 or fulvestrant for 5 days. At the end of treatment, surviving cells were counted and normalized to control cells that were treated with vehicle (DMSO) only. **(B)** IC_50_ values were obtained by deriving logarithmic curves from the %cell survival vs. treatment dose plot.

We have previously reported that ZB716 acted both as a strong antiestrogen and a potent ER degrader against T47D breast cancer cells with IC_50_ values comparable to fulvestrant [17]. Here we show that its action in MCF-7 breast cancer parallels that in T47D in terms of anti-proliferative and ER downregulation efficacies. ZB716 exhibited a dose dependent inhibition of MCF-7 cell growth with an IC_50_ measured at 3.2 nM, compared to fulvestrant at 1.5 nM (Figure 4A). As shown in Figure 4B, when MCF-7 cells were treated with ZB716 or fulvestrant for 4 hours and analyzed for ER expression level, downregulation of the hormone receptor occurred in a dose-dependent manner consistent with our previous observations with T47D cells [17].

**Figure 5:**
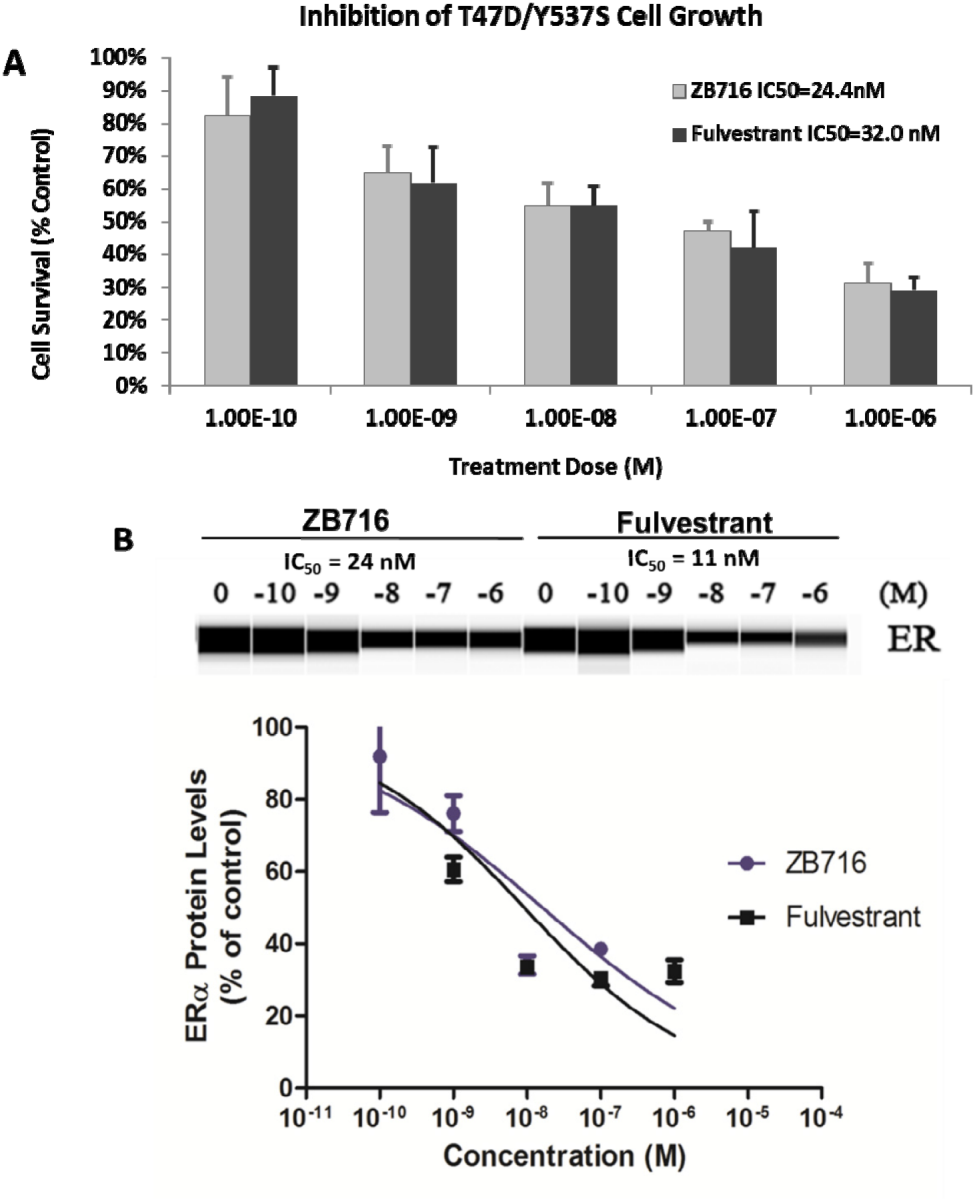
**(A)** T47D-Y537S breast cancer cells were treated with increasing doses of ZB716 or fulvestrant for 5 days. At the end of treatment, surviving cells were counted and normalized to control cells that were treated with vehicle (DMSO) only. IC_50_ values were obtained by deriving logarithmic curves from the %cell survival vs. treatment dose plot. **(B)** Dose-dependent ER downregulation in T47D/Y537S cells by ZB716 and fulvestrant.

To determine if ZB716 is effective as an antiestrogen in a clinically relevant breast cancer model that is estrogen independent and resistant to antiestrogens, we used an ESR1 mutant cell line, T47D/Y537S that was derived from a PDX model [26]. Y537S *ESR1* mutation has been found in recurring advanced breast cancer at high frequency [8, 9, 26, 27]. Cells were treated with ZB716 or fulvestrant at concentrations ranging from 0.1 nM to 1 μM. As shown in Figure 5A, ZB716 demonstrated a dose-dependent inhibition of growth; the IC_50_ for ZB716 and fulvestrant was found at 2.44×10^−8^ M and 3.20×10^−8^ M, respectively, about 10 times higher than in the T47D cells with wild type ER [17]. We next evaluated the ability of ZB716 to downregulate the mutant ER. In Figure 5B, downregulation of ER by 50% required approximately 10 times higher drug concentration, as reflected in the IC_50_ values, which are 24 nM for ZB716 and 11 nM for fulvestrant.

### ZB716 inhibits growth of MCF-7 human breast cancer xenograft in mice

**Figure 6:**
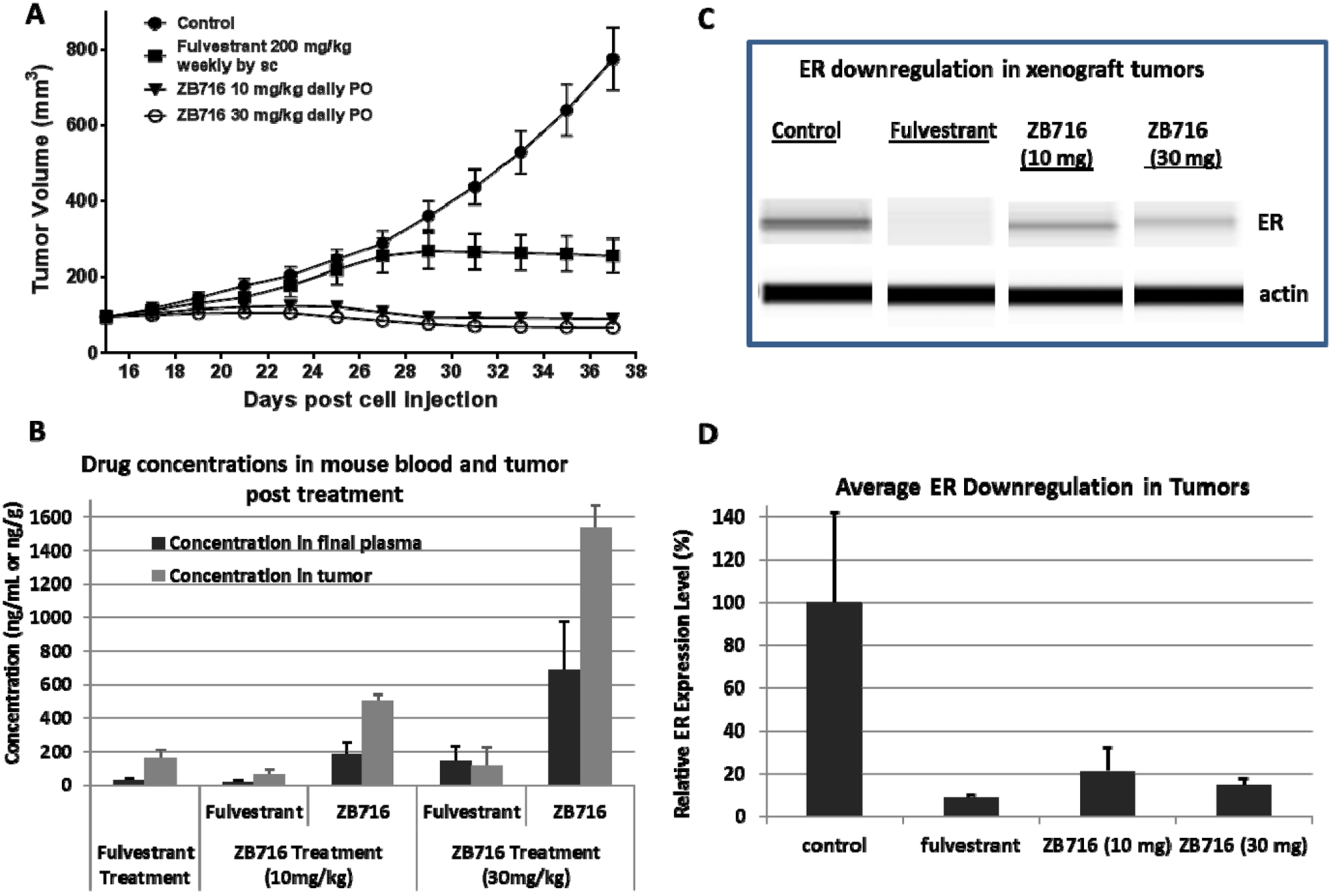
Nude mice bearing MCF-7 breast cancer xenograft were treated with either fulvestrant by s.c. injection or ZB716 at two different doses PO Treatment continued for three weeks before the animals were sacrificed and plasma and tumor tissues were collected. **(A)** tumor volumes were plotted vs. days of drug treatment; **(B)** concentration of ZB716 and fulvestrant in final plasma and tumor tissue samples at end of study; **(C)** WES analysis of ER expression in tumors collected at end of study; and **(D)** average ER expression in tumor tissues at end of study.

To test the efficacy of orally administered ZB716 *in vivo*, we used an MCF-7 human breast cancer xenograft model in nude mice. After the tumor formed and became palpable, the animals were randomized into four groups, and treated with vehicle, fulvestrant at 200 mg/kg weekly by subcutaneous (s.c.) injection, ZB716 at 10 mg/kg, or 30 mg/kg by oral gavage. Tumor sizes were monitored and recorded every other day for three weeks of treatment duration. As shown in Figure 6A, treatment with ZB716 resulted in complete blockage of tumor growth at both 10 mg/kg and 30 mg/kg, indicating that the lower dosage may have reached full therapeutic efficacy. We next measured the drug concentrations in the plasma and tumor tissues collected from mice euthanized at end of study. In the fulvestrant treatment group, the only active ingredient measured was fulvestrant, whereas in the ZB716 treatment groups, both ZB716 and fulvestrant were monitored as active ingredients. The average fulvestrant level in the final plasma of the fulvestrant treatment group was found at 27.92 ng/mL, whereas the average concentration of ZB716 and fulvestrant in the ZB716 treatment groups were measured at 181.96 ng/mL and 18.35 ng/mL for 10 mg/kg dose, at 691.88 ng/mL and 144.6 ng/mL for 30 mg/kg (Table 1). As previously noted, fulvestrant is an active metabolite of ZB716, constituting about 10-20% of total active ingredients in mice plasma [17]. In the 10 mg/kg treatment group, the final plasma concentration of ZB716 reached 181.96 ng/mL after continuous oral administration of the drug, reflective of a steady-state level that primarily accounts for the superior efficacy of ZB716 as compared to fulvestrant treatment. At the dose of 30 mg/kg ZB716, plasma levels of both ZB716 and fulvestrant were further increased, ensuring maximal inhibition of tumor growth in mice. We also observed that in tumor tissues, accumulation of drugs resulted in a 2-3 fold higher concentrations than in plasma, significantly higher than therapeutically effective levels in breast cancer cells (Table 1 and Figure 6B). In addition, tumor tissues were also analyzed for ER expression level. Figure 6C and 6D show the downregulation of ER in tumors from three treatment groups of mice.

**Table 1:** Drug distribution in plasma and tumor tissues

Treatment	Fulvestrant (200 mg/kg/wk)	ZB716 (10 mg/kg daily)		ZB716 (30 mg/kg daily)	
	Fulvestrant	Fulvestrant	ZB716	Fulvestrant	ZB716
Average concentration in final plasma (ng/mL)	27.92	18.35	181.96	144.6	691.88
SEM	11.48	11.55	71.72	90.24	283.71
Average concentration in tumor tissue (ng/mL)	159.6	67.87	506.1	118.5	1539.67
SEM	47.6	27.03	34.41	109.13	129.33

### ZB716 inhibits growth of patient-derived xenograft (PDX) tumor in mice

**Figure 7:**
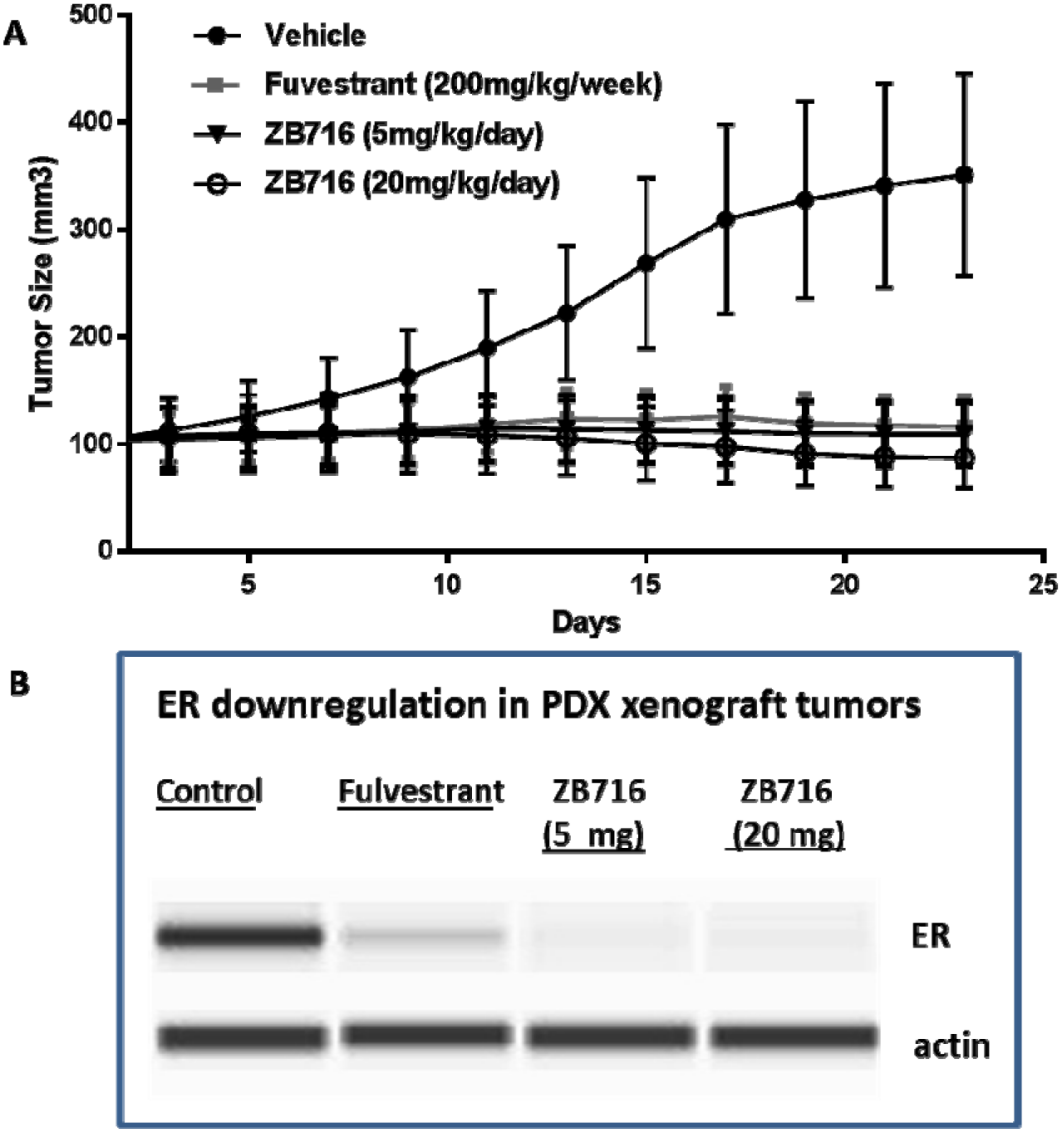
**(A)** Inhibition of PDX breast tumors by ZB716 orally administered to mice at 5 and 20 mg/kg, and by fulvestrant 200 mg/kg subcutaneously injected at 200 mg/kg weekly. **(B)** Downregulation of ERα in tumor tissues treated by fulvestrant, ZB716 5mg/kg, or ZB716 20 mg/kg, respectively.

The efficacy of orally administered ZB716 was next evaluated in a patient-derived xenograft mouse model in which the primary tumor donated by a postmenopausal patient was engrafted in NOD *scid* gamma (NSG^™^) mice (TM00386 PDX model, Jackson Lab). This model has been immunohistochemically confirmed as ER+/PR+/HER2- invasive ductal carcinoma. PDX tumor bearing mice were treated with vehicle, fulvestrant 200 mg/kg by weekly s.c. injection, ZB716 at 5 mg/kg PO daily, or ZB716 at 20 mg/kg PO daily. As shown in Figure 7, ZB716 at both doses were effective in blocking tumor growth in the PDX mice, with the 20 mg/kg treatment group showing the greatest effect on tumor growth inhibition. Notably, the lower dose of 5 mg/kg demonstrated *in vivo* efficacy in blocking PDX tumor growth as effectively as fulvestrant treatment.

### ZB716 demonstrates a dose-dependent oral bioavailability

When ZB716 was administered to Sprague Dawley rats at increasing doses, plasma concentrations of ZB716 and its active metabolite, fulvestrant, were measured at various time points in plasma samples. As shown in Table 2, for all four doses, ZB716 reached peak concentrations in rat plasma at 1 hour after dosing, and the C_max_ level increased proportionally with dose levels. Notably, the peak concentration of ZB716 observed in the 20 mg/kg dosage reached over 1000 ng/mL in rat plasma, indicating sustainable high oral bioavailability at elevated doses.

**Table 2:** Dose-dependent pharmacokinetics of ZB716 orally administered to rats

Time (hrs)	Rat plasma concentration (ng/mL) of ZB716 after a single oral dose			
	1 mg/kg	5 mg/kg	10 mg/kg	20 mg/kg
1	14.85±6.51	46.52±4.81	217.66±27.76	1044.53±153.17
3	9.23±2.33	23.37±2.80	93.58±21.77	670.93±125.16
6	4.70±1.66	15.92±2.03	62.04±14.86	370.93±111.46
8	4.42±2.38	11.42±0.52	60.74±13.93	299.84±118.47
24	1.36±1.42	9.37±1.24	33.09±10.58	98.53±36.11

## DISCUSSION

Clinical challenges facing the current SERD regimen for breast cancer therapy include low response rate, poor bioavailability, and slow action associated with intramuscular injection-only route of administration as a 500 mg/10mL oil-based depot. These limitations of fulvestrant have driven an intensifying race to a clinically proven, orally bioavailable SERD. At least five non-steroidal SERD candidates have entered phase I clinical trials since 2014, yet none has advanced to phase II trials except Genentech's GDC-0810, which unfortunately was recently discontinued from an ongoing phase II clinical trial due to G.I toxicities. This latest development highlights the uncertain clinical path for nonsteroidal oral SERDs. Therefore, in order to achieve clinical benefits beyond the currently approved fulvestrant, an orally bioavailable SERD must satisfy clinical criteria including fast action, more durable treatment outcome by virtue of high drug exposure, and tolerable adverse side effects. Design and development of ZB716 represents a unique and promising approach in that it remains as a steroidal SERD with minimal structural modifications to vastly improve its oral bioavailability. After initially demonstrating its *in vitro* mode of action as a pure antiestrogen and ER downregulator in ER+ breast cancer cells and excellent oral bioavailability in mice [17], we now report further studies of the compound to compare with fulvestrant in binding to ER and its efficacy as an oral SERD.

The modeling study of ZB716's binding mode to ERα revealed details of the molecular interactions of ZB716 with the ER ligand binding domain that are nearly identical to those of fulvestrant. The docking results are helpful in understanding the small differences between ZB716 and fulvestrant in their *in vitro* activities against breast cancer cells observed earlier [17], and provide further evidence that replacement of the 3-hydroxyl group with a boronic acid moiety does not change its binding behavior. As a steroidal SERD structurally analogous to fulvestrant which has well-established side effect profiles, ZB716 may face less toxicological uncertainties in clinical trials than the non-steroidal SERDs currently under evaluations in patients.

Receptor conformation dictates affinity for and recruitment of coregulator proteins to the target gene. These coregulators can alter local chromatin accessibility for the transcription machinery and eventually determine target gene expression levels. Therefore, similarity of *in vitro* coregulator recruitment profiles between ZB716 and fulvestrant is strongly correlated with similar modes of action and pharmacology *in vivo*. PK/PD factors play additional roles in *in vivo* drug effectiveness, which will give ZB716 the competitive edge on top of the effective pharmacology that is shared with fulvestrant.

The finding that a higher dose of either fulvestrant or ZB716 is required to inhibit the growth of ER mutant (Y537S) breast cancer cells underscores the risk of insufficient drug exposure in the clinical setting where fulvestrant is used to treat recurring diseases that harbor ER mutant variants. Consistent with ZB716's performance in other ER+ breast cancer cells, the compound was similarly effective in downregulating ER in both breast cancer cells with wild type ER and those with mutant ER (Figure 5) when compared with fulvestrant. These reproducible characteristics of ZB716 in turn are anticipated to be translatable to enhanced *in vivo* efficacy owing to its high oral bioavailability as seen in mice [17].

Indeed, the first *in vivo* experiment using an MCF-7 mouse xenograft model demonstrated the superior efficacy of ZB716 in blocking tumor growth (Figure 6). Compared to fulvestrant, which was given as a standard 200 mg/kg weekly injection, ZB716 was more effective at both treatment doses of 10 mg/kg and 30 mg/kg orally. Given that ZB716 has been consistently shown to be slightly less potent than fulvestrant in cellular assays and binding assays, its greater *in vivo* efficacy is most certainly attributable to the markedly higher bioavailability of ZB716. This assumption was further confirmed by the final plasma concentrations of ZB716 in mice from both dose groups where drug concentrations were found to be over 6- and 20-fold higher than in the fulvestrant treatment group. Moreover, when tumor tissues were analyzed for drug concentrations, we note that ZB716 afforded a 4-fold higher drug accumulation level for the 10 mg/kg dose group, and 10-fold higher drug concentration for the 30 mg/kg dose group, than in the fulvestrant treated mice. We note that the average downregulation of ER in tumor tissues did not appear to be fully in line with efficacy, possibly due to the effect of circulating estrogen (from implanted E2 pellets) that suppresses ER expression to various degrees in tumor tissues. The oral efficacy of ZB716 was further verified in the second *in vivo* experiment using a PDX mouse model hosting an ER+/PR+/Her2- primary breast tumor (Figure 7) where both ZB716 dose groups showed regression of tumor comparable to the fulvestrant group. Importantly, in all tumor bearing animals treated with different oral doses of ZB716, no apparent toxicities were observed in the entire course of treatment, and no significant loss or increase of animal body weights was recorded.

An important question to be answered with regard to the oral bioavailability of ZB716 is whether its systemic circulation level is dose-dependent. This is highly relevant to both first-line and second-line settings in the clinic. In the former, dose optimization to achieve therapeutically effective drug levels in patients’ systemic circulation with manageable adverse side effects is critical for a long-term first line regimen, and dose-dependent oral bioavailability enables such optimization. In the latter when ZB716 is potentially indicated for second- or third-line endocrine treatment, higher drug exposure may be necessary for rapid response and maximal clinical benefits in advanced breast cancer patients. A four-dose pharmacokinetic study (Table 2) demonstrated a favorable dose-response of ZB716's oral bioavailability. The 20 mg/kg dose afforded a peak plasma concentration of over 1000 ng/mL, or 1.6 μM, a level that far exceeds the therapeutically effective concentration of a potent SERD with low nanomolar IC_50_ values in endocrine sensitive breast cancer cells, and a level that is also significantly higher than its IC_50_ value in ER mutant breast cancer cells.

## MATERIALS AND METHODS

### Molecular modeling

To study interactions of ZB716with ERα, computational docking and modeling studies were performed using the Glide program in Schrodinger Suite 2015-3. The antagonistic ligands induce conformational changes on ER_α_ and prevent the binding of coactivator signal transmitting proteins, thus impair hormone dependent ER transactivation [28]. Since both fulvestrant and ZB716 act as ER antagonists, the X-ray crystal structure of ERα in complex with an antagonist (6-(4-methylsulfonyl-pheynyl)-5-[4-(2-piperidin-1-ylethoxy)phenoxy]naphthalene-2-ol) with a resolution of 1.9 Å (PDB entry: 2AYR) was used for binding studies. The initial 3D coordinates for ERα was prepared by removing all the crystallographic water molecules beyond 5 Å from the crystal ligand and adding hydrogen atoms consistent with physiologic pH of 7 using Maestro 10.3. Then the protein molecule was energy minimized with an RMSD cutoff value of 0.3 Å for all heavy atoms. The ligand molecule, fulvestrant, was prepared by optimizing the initial coordinates obtained from the ZINC database using Maestro. The structure of ZB716 was prepared by replacing the 3-hydroxyl group with a boronic acid group followed by energy minimization. The binding site for antagonists on the ERα is well characterized by the crystallographic studies of ERα in complex with various antagonists [21–24]. Thus the antagonist binding site-based receptor grid was generated for docking with the ER. The ER-ligand docking was then performed with Glide 6.8 using default parameters under the extra precision (XP) mode allowing the procurement of the best docked representative structure. Finally, the binding free energies of the complexes were calculated using the MM/GBSA method with OPLS/AA force field and a GB/SA continuum solvent model.

### Compound-mediated modulation of ERα-coregulator interaction

The modulation of ERα-coregulator interaction by compound was analyzed using MARCoNI (Micro Array Assay for Real-time Coregulator Nuclear receptor Interaction) as described previously [29] using 7 nM GST-tagged ERα LBD (Invitrogen).

In short, a reaction mix with ERα LBD and fluorescently labeled detection antibody with 10 μM of the indicated compound or solvent (DMSO, 2% final concentration) only is incubated on a microarray containing 154 coregulator-derived NR-binding motifs. Each condition is measured using 3 technical replicates (arrays). After incubation, unbound receptor is removed by washing, and a tiff image of each array is acquired using a CCD camera and receptor binding to each peptide on the array is quantified using dedicated software. For each condition, the three technical replicates are used to calculate mean and S.E.M. ERα binding as well as compound-induced log-fold modulation vs. control for each individual motif. Significance of the modulation is assessed using Student's t-Test.

### T47D Y537S ER mutant cell line

Wild-type ESR1, the Y537S ESR1 mutant were fused to a FLAG tag at their C-termini and cloned into the lentiviral vector pFLRu-FH [30]. Y537S mutation was first introduced into ESR1 using the QuikChange II XL Site-Directed Mutagenesis Kit (Agilent) with an ESR1-encoding plasmid (Accession number NM_000125.1, GeneCopoeia Inc.) as the template. A FLAG-tag (DYKDDDDK) was then added to the C terminus of full-length wild-type and mutant (Y537S) ESR1 by PCR amplification, followed by shuttling into the designation lentiviral vector pFLRu-FH. To make lentiviral particles, pFLRu-FH vector DNAs (encoding ESR1(wt) and ESR1(Y537S)) were cotransfected with the packaging plasmids into HEK293T cells using Fugene 6 (Roche). At 48 hours post transfection, culture media containing different viruses were added to T47D cells in the presence of polybrene followed by 3-day puromycin selection for stable expression. Transgene expression was verified by western blot analysis for wild-type and mutant ER mutant proteins.

For growth assays in the presence of ZB716 or fulvestrant, T47D Y537S ESR1 mutant cells were plated in six-well plates at a density of 50,000 each well in 5% FBS DMEM medium. The cells were then treated with ZB716 or fulvestrant at 5 different doses ranging from 10^−10^ M to 10^−6^ M for 5 days, while equal volumes of DMSO were used as vehicle controls. Viable cell numbers were counted with a Z Series Coulter Counter instrument (Beckman-Coulter) following manufacturer's instructions. The ratio of drug treated viable cell numbers to vehicle treated viable cell numbers was defined as survival ratio where the control has the survival ratio of 100%. IC_50_ values were obtained from dose-response curves for all treatments.

### Western blot of ER downregulation

MCF-7 cells were plated at a density of 200,000 cells/60mm dish. Media containing the same drug concentrations as the growth curve assay were added on the day following plating (day 0) and allowed to incubate for 5 days for Western blot. Media with the tested compound was changed every other day. Cells were lysed. Lysates were placed on a rotisserie at 4°C for 30 min and then spun at 4°C at 12,000 rcf for 10 min. Supernatants were assayed for protein content, snap-frozen, and stored at −80°C if not run immediately. 50 μg of protein was subjected to Western blot protocol. Membranes were blocked and then incubated with 1:200 dilution of ERα antibody at 4°C overnight followed by 1:10,000 dilution of secondary antibody for 1 hr at room temperature. They were then imaged on a LICOR infrared scanner.

Total ERα protein levels in T47D Y537S ER mutant cells were determined using automated Western blotting (Wes Simple Western Analysis, ProteinSimple, San Jose, CA). Simple Western analyses were performed according to the ProteinSimple user manual. Briefly, T47D Y537S ER mutant cells were grown in charcoal stripped serum for 6 days and plated at a density of 250,000 cells per well in a 12-well plate. Cells were treated with either DMSO, ZB716, or fulvestrant at the indicated concentrations for four hours, and protein was extracted from cells using MPER lysis buffer (Pierce) containing protease and phosphatase inhibitors (Thermo Scientific). Samples were mixed with a master mix (ProteinSimple) to give a final concentration of 0.2 mg/mL total protein, 1×sample buffer, S61×fluorescent molecular weight markers, and 40 mM DTT. Samples were heated at 95°C for 5 min followed by centrifugation. Samples, blocking solution, primary antibodies, horseradish peroxidase-conjugated secondary antibodies, chemiluminescent substrate, and separation and stacking matrices were loaded into designated wells in a 384 well plate. After plate loading, fully automated electrophoresis and immunodetection took place in the capillary system. Proteins were separated by molecular weight at 375V for 25 min, and primary and secondary antibodies incubated for 30 minutes. The ERα (Santa Cruz, cat # sc-543) and actin (Novus, cat #NB600-503) antibodies were diluted in a proprietary antibody diluent at a 1:50 dilution ratio. Chemiluminescence was captured by a charge-coupled device camera, and the digital image was analyzed using Compass software (ProteinSimple). The relative amount of each protein, relative to total protein content, was calculated based on peak area. Total ERα levels were normalized to actin. IC50 values were obtained from dose-response curves for all treatments.

### Efficacy study in an MCF-7 xenograft tumor model

Four to six weeks old female ovariectomized Nu/Nu mice were purchased from Charles River Laboratories (Wilmington, MA). MCF-7 cells were cultured and harvested in the exponential growth phase using a PBS/EDTA solution. The animals were injected bilaterally in the mammary fat pad (MFP) with 5×10^6^ viable cells suspended in 50 μL sterile PBS mixed with 100 μL Matrigel (reduced factor; BD Biosciences, Bed- ford, MA). 17b-Estradiol pellets (0.72 mg, 60 day release; Innovative Research of America, Sarasota, FL) were implanted subcutaneously in the lateral area of the neck using a precision trochar (10 gages) at the time of cell injection. Tumors were allowed to form and at day 15 post cell injection mice. After the tumor formed and became palpable, the animals were randomized into four groups, and treated with vehicle, fulvestrant at 200 mg/kg weekly by subcutaneous injection, ZB716 at 10 mg/kg, or 30 mg/kg by oral gavage. Tumor sizes were monitored and recorded every other day for three weeks of treatment duration.

### Efficacy study in a patient derived xenograft (PDX) tumor model

In a patient-derived xenograft (PDX) mouse model in which the primary tumor donated by a postmenopausal patient was engrafted in NOD *scid* gamma (NSG™) mice (TM00386 PDX model, Jackson Lab). After the tumor formed and became palpable, the animals were randomized into four groups, and treated with vehicle, fulvestrant at 200 mg/kg weekly by subcutaneous injection, ZB716 at 5 mg/kg, or 20 mg/kg by oral gavage. Tumor sizes were monitored and recorded every other day for three weeks of treatment duration.

### Pharmacokinetic study (sampling and analysis)

Female Sprague-Dawley rats, weighing between 350 and 400 g (Charles River Laboratories, Portage, MI) were used for the pharmacokinetic study on ZB716. Rats were given oral gavage containing 5% dimethyl sulfoxide (DMSO), 40% polyethylene glycol 400, 55% saline-dissolved ZB716, at doses ranging from 1 mg/kg to 20 mg/kg. After drug administration, blood samples were collected from the lateral tail vein of the rat at 1, 3, 6, 8, 24 h. Rat blood was collected with a capillary into 1.5 mL microcentrifuge tubes containing 0.01 mL of 10 % EDTA anticoagulant. Plasma was then separated from cell pellets by centrifugation in a refrigerated centrifuge at 4°C and transferred to a separate tube. Plasma samples were frozen at −80°C until analysis.

The plasma samples of 100 μL each were processed for analysis by a TSQ UPLC-MS/MS system. Aliquots of 10 μL of 0.5 ng/μl E-Tamoxifen-2C^13^, N^15^, 200 μL methanol, and 400 μl chloroform were added to the plasma in a 1.5 mL centrifuge tube in sequence. The sample was vortexed and stored at −20°C for 4 hours, followed by sonication in a Branson B3510MT Ultrasonic Cleaners for 30 min and centrifuged at 12000 RPM on a Heraeus Fresco 21 Refrigerated Micro Centrifuge from Thermo Fisher Scientific for 10 min. The supernatant was dried with a nitrogen gas flow and suspended in 100 μL methanol. Aliquots of 10 μL of the above processed samples were injected into a TSQ Vantage mass spectrometer connected with a heated electrospray ionization probe and a Dionex Ultimat 3000 UHPLC with a Hypersil gold column (50×2.1 mm). The probe setting and front mass spectrometer setting were controlled as follows: spray voltage 3200 voltage, auxiliary gas 10psi, vaporizer temperature 365°C, capillary temperature 300°C, sheath gas 30psi, S-lens RF amplitude 51 voltage. The flow gradient was started with initial 30% mobile phase A of water with 0.05% formic acid to 1 min, to 100% mobile phase B of acetonitrile with 0.05% formic acid at 8 min, and return to 30% mobile phase A at 13 min.

## SUPPLEMENTARY MATERIALS AND FIGURES


