# Supplementary material for: ZB716, a steroidal selective estrogen receptor degrader (SERD), is orally efficacious in blocking tumor growth in mouse xenograft models
Source: Oncotarget. 2018 Jan 8;9(6):6924–37. doi: 10.18632/oncotarget.24023 (PMC5805526; doi:10.18632/oncotarget.24023)
Supplement: Supplementary file 1 [file oncotarget-09-6924-s001.pdf]

ZB716, a steroidal selective estrogen receptor degrader (SERD), is orally efficacious in blocking tumor growth in mouse xenograft models

SUPPLEMENTARY MATERIALS

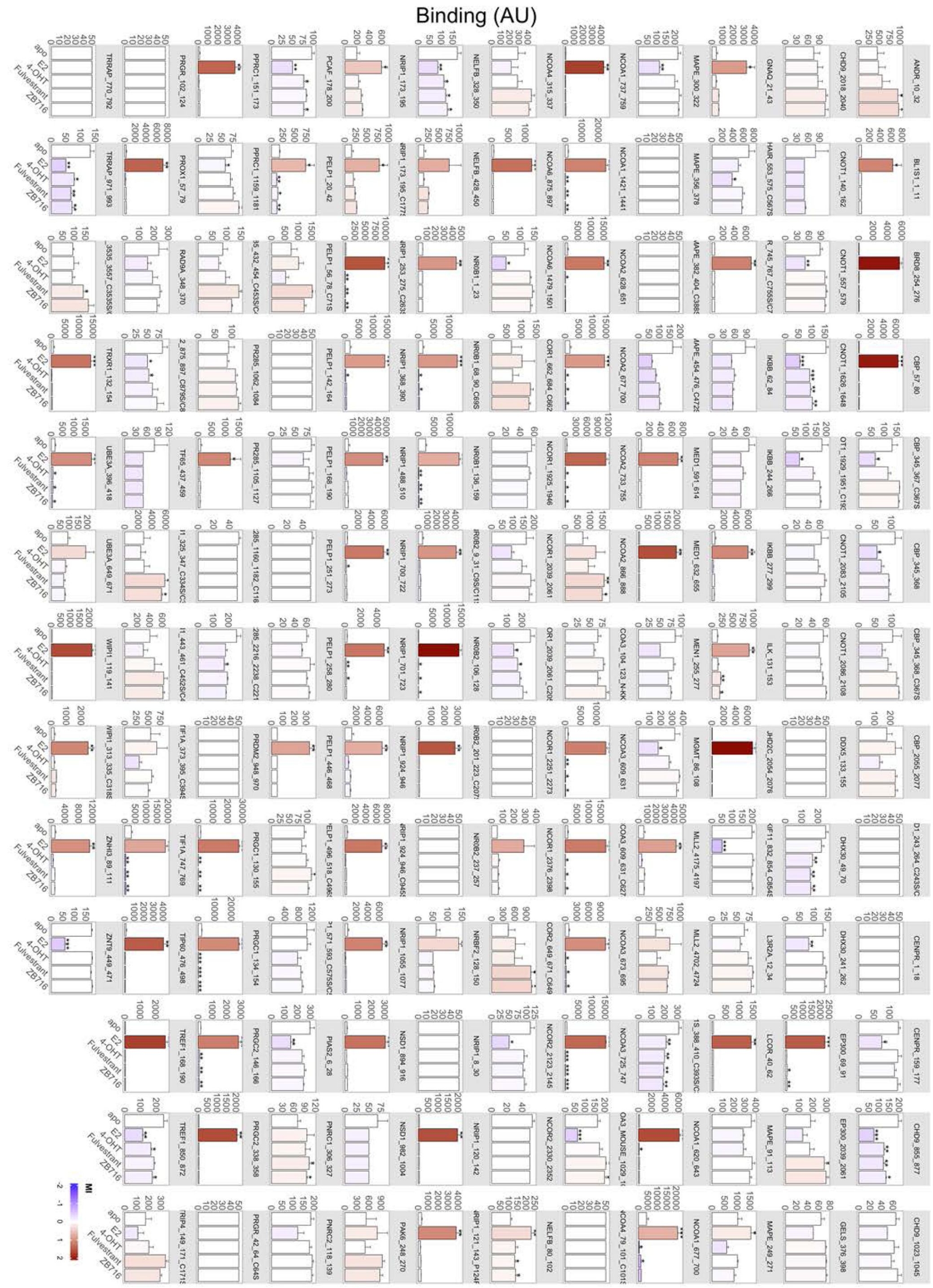

Supplementary Figure 1: Bargraphs of ER interactions with coregulators in absence (apo) or presence of indicated compound.
